# Return-to-Play Protocols for Various Injuries in Skeletally Immature Athletes: A Systematic Review

**DOI:** 10.7759/cureus.106148

**Published:** 2026-03-30

**Authors:** Delaney Tyl, Anastasia Laurenzo-Wrigley, Christopher Rennie, Katerina N Futch, Elena Arcaroli, Alessandra Ottley, Melissa K Rodriguez, Mackenzie Elting, Leighann C Krasney

**Affiliations:** 1 College of Medicine, Nova Southeastern University Dr. Kiran C. Patel College of Osteopathic Medicine, Tampa, USA; 2 Orthopaedic Surgery, Philadelphia College of Osteopathic Medicine, Philadelphia, USA; 3 College of Medicine, Nova Southeastern University Dr. Kiran C. Patel College of Osteopathic Medicine, Fort Lauderdale, USA; 4 College of Medicine, Lincoln Memorial University-DeBusk College of Osteopathic Medicine, Harrogate, USA; 5 Shoulder and Elbow Orthopaedic Surgery, Mountain Valley Orthopedics, Stroudsburg, USA

**Keywords:** pediatric trauma, return to play, sports injury, sports medicine, youth sports

## Abstract

Sports-related injuries are a commonly encountered orthopedic complaint in the United States. Return-to-play (RTP) protocols are in place to guide safe progression through injury recovery and offer a stepwise timeline for resuming athletic activity. In pediatric athletes, this is increasingly important in the context of disrupted growth, muscular and skeletal imbalances, and heightened social importance of team-based activities. Despite this, there is limited literature defining a concise RTP protocol for pediatric athletes suffering from various common sports-related injuries. The primary aim of this investigation was to systematically review and synthesize existing RTP protocols for the most commonly encountered pediatric athletic injuries based on the currently available literature at this time. The literature search for this analysis was performed in the Biomedical Reference Collection, Cochrane Library, CINAHL, and MEDLINE. After inclusion criteria were met and article screening was performed, a total of 36 articles were selected for further qualitative analysis. This review reaffirmed the lack of standardized RTP protocols for pediatric sports injuries. A total of 51% of the protocols analyzed involved anterior cruciate ligament (ACL) tears and concussions, both of which hold highly variable RTP guidelines. For ACL injuries, this review determined an average of 9-12 months of recovery is the most commonly utilized timeframe; however, younger athletes may require upwards of 14-16 months depending on physeal maturation. While concussion protocols are relatively accepted across athletic disciplines, there are possible improvements available, including sport-specific testing such as the Gapski-Goodman Test, provocation tests, or ImPACT scoring. RTP protocols are highly variable depending on a variety of factors such as the type of provider performing the assessment, age of the patient, and the sport involved. Further research is necessary to further delineate the nuanced differences in safe RTP guidelines for injuries seen in each sport.

## Introduction and background

Return-to-play (RTP) decisions are essential to treating injuries in sports medicine, especially when skeletally immature athletes are involved. These protocols safely guide the step-by-step progression of activity during the recovery from an injury as determined by the healthcare provider. Essential factors must be considered, such as the patient’s age, their functional capabilities, the severity of the injury, the level and functional requirements of the specific sport, athlete safety, and potential risk to the safety of other participants, and governing body regulations [1]. RTP protocols exist for a variety of injuries, with the most well-researched recovery after anterior cruciate ligament (ACL) reconstruction surgery and following concussions.

Decisions regarding RTP include four major facets. The first is the medical component, which is the least clearly defined. This uncertainty is due to gaps in knowledge regarding specific pathophysiology and natural disease progression that exist for every injury [1]. As knowledge is advanced by further research, the protocols are updated, making them ever-changing and difficult to follow. The three other facets of RTP include social and economic resources, politics regarding who is in the position to decide when to resume activities, and legal and legislative issues [1].

The treating physician or primary care provider has traditionally made decisions regarding RTP. Over the years, many other qualified healthcare professionals have become involved in the treatment of sports injuries. These include physical therapists, athletic trainers, and strength and conditioning coaches. Although the involvement of all these specialties within an athlete's sport is of significant benefit to them, it has also led to potential confusion regarding the decision-making process. Since each specialty has begun researching its own RTP protocols, this has led to disarray about which protocol to follow. The lack of standardization can be a source of disagreement for physicians, athletes, coaches, and administrators [2].

The specific decisions regarding progression vary greatly between medical conditions and various circumstances and are thus not a one-size-fits-all solution. When choosing criteria for RTP, prioritizing safety is preferred, but being overly conservative without compelling evidence can lead to unnecessary restriction of patients from sports and activities [3]. RTP protocols often include steps that take the patient from no activity through sport-specific exercise and back to full play. Many variations of RTP protocols are adapted for different sports, but these steps outlined by the Centers for Disease Control and Prevention's (CDC) Heads Up Return to Play guidelines following concussions are a typical example of most protocols. The six steps are as follows: (1) return to regular daily activities such as school, (2) light aerobic activity such as 5-10 minutes on an exercise bike or walking, (3) moderate intensity activity such as jogging or moderate intensity weightlifting, (4) heavy, non-contact activity such as sprinting and non-contact sports specific drills, (5) practice and full contact, (6) return to competition. Each step should take at least 24 hours. The athlete is monitored for their ability to tolerate the increased activity and whether symptoms return or new symptoms develop during each step [4]. These protocols can become more complex when applied to injuries other than concussions and to sports that require more contact during games. RTP guidelines should also consider the treatment method being used, such as conservative versus operative management.

RTP protocols in pediatric sports medicine have distinct challenges that need to be addressed. The RTP decision in the pediatric population is complicated by not only the patient and their desires, but also the opinions of their parents, coaches, teammates, and other healthcare providers. Ensuring clear communication among all parties involved in a child athlete’s care is imperative in RTP progressions. While it is beneficial to have an ultimate decision-maker, a collaborative decision-making processes that involve all members of the medical team can yield advantageous outcomes [2]. Another unique complication in the pediatric population is that previous injury is associated with a significant increase in the risk of sustaining a similar injury [5]. Specifically in pediatric sports medicine, it is noted that reinjury rates following initial injuries are high, averaging between 15% and 32% and increasing with age [3]. The prevention of reinjury is highly dependent on when it is safe to resume full sports participation.

Increased media coverage of the consequences of poorly managed injuries, such as concussions, as well as a history of decision-making based exclusively on experience and expert opinion, has led to the development of evidence-based RTP protocols [3]. A push to create these protocols has led to some standardization for specific injuries, but many remain inconsistent. Few published articles provide a comprehensive view of current RTP protocols for the most common sports injuries. This review aims to address this gap in the current literature by systematically synthesizing current evidence-based RTP protocols for the most common pediatric athletic injuries reported in applicable peer-reviewed studies.

## Review

Methods

Article Eligibility Criteria

**Figure 1:**
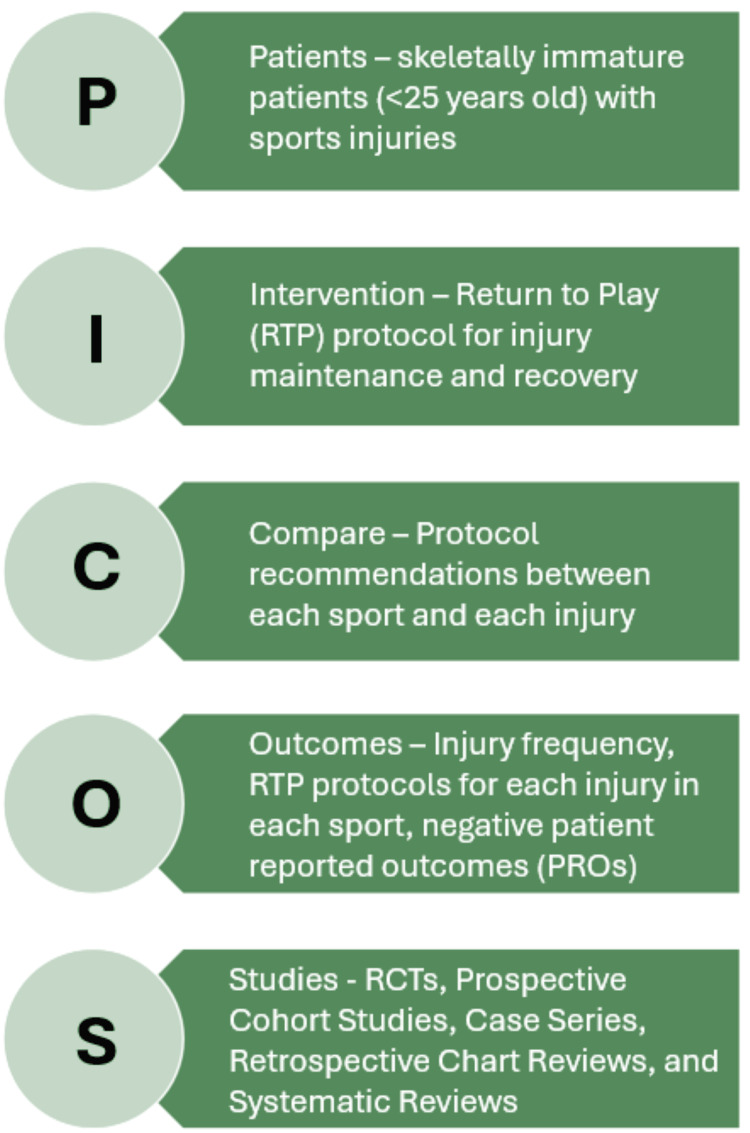
PICOS criteria for article eligibility PICOS: Patients, Intervention, Compare, Outcomes, and Studies; RCT: randomized controlled trial Image credit: Christopher Rennie

Articles were deemed eligible based on the PICOS framework of article characteristics: Patient, Interventions, Comparative Interventions, Outcomes, and Studies. Inclusion criteria utilizing the PICOS method are detailed in Figure 1. Additionally, only articles published or available in English were included. This study placed no limit on publication date, national origin, type of sport, or type of injury. Studies including athletes under 25 years of age were eligible for inclusion. Although skeletal maturity is typically achieved earlier, many sports medicine studies group adolescent and young adult athletes together and do not consistently report physeal status. Therefore, an upper age limit of 25 years was selected to capture literature relevant to high school and collegiate-aged athletes, who are frequently managed within similar RTP frameworks. When reported, skeletal immaturity or physeal status was prioritized in data interpretation.

Search Strategy and Terminology

The following key terms and boolean operators were utilized in the initial literature search: “return to play” OR “return to sport” OR “return to participation” OR “RTS” or “RTP” AND “pediatrics” OR “Children” OR “child” OR “kids” OR “youth.” In March of 2024, this search string was entered in each of the following databases: Biomedical Reference Collection, CINAHL, Cochrane Library, and PubMed/MEDLINE.

Article Screening and Selection

This review was conducted in accordance with the Preferred Reporting Items for Systematic Reviews and Meta-Analyses (PRISMA) guidelines. Once articles were identified using the search strategy detailed above, three reviewers independently screened the studies for the aforementioned PICOS criteria. Initial screening involved automatic duplication removal, followed by exclusion based on title relevance, abstract appraisal, and full article assessment. After blind review, if any discrepancies in article inclusion were noted, these were discussed and agreed upon with a third party prior to qualitative analysis. Additionally, methodological quality and potential sources of bias were evaluated independently by reviewers during the screening process using predefined inclusion and exclusion criteria based on the PICOS framework. Study design, sample size, clarity of RTP criteria, and completeness of reported outcomes were considered when determining study eligibility. A formal, validated risk-of-bias scoring tool was not applied. If an article failed to meet the inclusion criteria at any step of the screening process or demonstrated substantial methodological limitations during reviewer assessment, the study was excluded from further analysis.

Data Extraction and Analysis

Once article screening was complete, the following information from each study was extracted: author information, publication date, national origin, study design, patient characteristics, sports and injuries evaluated, main outcomes, and the general RTP protocol recommendation. This information was recorded as seen in Table 1 and utilized for analysis. Due to significant heterogeneity in injury type, study design, RTP criteria, and outcome reporting, a quantitative meta-analysis was not feasible, and findings were synthesized descriptively.

**Table 1:** Summary of articles included in qualitative analysis ACL: anterior cruciate ligament; ACLR: anterior cruciate ligament reconstruction; GGT: Gapski-Goodman Test; MPFL: medial patellofemoral ligament; MTJ: myotendinous junction; MVPA: moderate vigorous physical activity; OCD: osteochondritis dissecans; ORIF: open reduction internal fixation; PCSI: post concussion symptom inventory; PROMS: patient-reported outcome measures; PT: physical therapy; ROM: range of motion; RTC: rotator cuff tear; RTP: return to play; RTS: return to sport; UK: United Kingdom; USA: United States of America; WB: weightbearing; YA: young adult

ACL Reconstruction							
Study	Country	Study Design	Patients	Sports Evaluated	Injuries Evaluated	Main Results	RTP Protocol Recommendations
Burland et al., 2018 [6]	USA	Retrospective cross-sectional	n=50, <19yo	Unspecified	ACL Reconstruction	Increased quadricep isometric and isokinetic strength at three and six months post-ACLR associated with earlier RTP	Higher strength measures at both three and six months after ACLR were associated with greater self-reported knee function and greater readiness to return to functional activities at six months and ultimately earlier return to sport in adolescent athletes
Butler et al., 2024 [7]	USA	Multicenter retrospective cohort	n=289, mean age 16yo	Soccer (32.9%), football (22.5%), basketball (18.7%), volleyball, baseball/softball	Primary ACL Reconstruction	No difference to RTP based on age. Males complete RTP testing sooner and females require more PT visits but no clinically meaningful difference	Average RTP testing conducted at eight months, with continued recommendation of nine months for clearance. In a youth cohort, age and sex may not have a clinically important effect on physical therapy utilization
Chicorelli et al., 2015 [8]	USA	Retrospective case series	n=250 <14yo, two years post-op	Unspecified	ACL Reconstruction	96% of athletes <14yo were able to RTP at the same skill level	50% of patients RTP by nine months, with 85% returning by 12 months. Consider emphasizing sport-specific rehab and sports psychology in those who did not return to sport
Cordasco et al., 2019 [9]	USA	Case series	n=324, <20yo	Soccer, football, skiing, lacrosse, basketball, nonathletic/ other sports	ACL Reconstruction	Patients who bridged late middle and early high school were the group with the highest revision rate and lowest RTS rate (compared to group 1 and 3 which were the youngest and oldest group, respectively). Younger athletes who undergo an all epiphyseal ACLR with a hamstring autograft appear to have a lower reinjury rate than older athletes treated with a partial transphyseal or complete transphyseal hamstring autograft technique	Late middle school and early high school-aged athletes are considered increased risk of revision surgery and lower return to sport. Risk profile may be used to counsel athletes and parents preoperatively regarding the expectations of surgery
Dekker et al., 2017 [10]	USA	Retrospective review	n=112, <18yo	Basketball, soccer, baseball/softball, volleyball, lacrosse, other	ACL Reconstruction	91% of subjects RTP, with the remaining expressing they did not return due to personal preference, not physical limitation. None were cleared to RTP by the surgeon before six months. Only statistically significant factor for reinjury was time to RTP	Patients should not RTP before six months to protect against reinjury
Dietvorst et al., 2020 [11]	Netherlands	Scoping review	n=26 studies, Number of patients unspecified, <18yo	Unspecified	ACL Reconstruction	Analysis of studies were organized into tests used to evaluate patients for RTS and times at which they are used. Tests include muscle strength tests, hop tests, movement quality, PROMS, physical exam testing. Seven studies included definitions of RTS clearance criteria	RTP testing for 16-18 yo should include: quad and hamstring test, hop tests, movement quality assessment during sport-specific tasks, PROMS. RTP testing for 12-16yo should include: hop tests, movement quality and pediatric PROMs
Geffroy et al., 2018 [12]	USA	Retrospective study	n=278, <18yo	Unspecified	ACL Reconstruction	RTS results were similar in children and adolescents. About 80% RTS after ACLR, 60% of which returned to the same or higher level. RTP time was long in both groups, over 10-12 months (over one year in young children). Retear rate was high especially in the group with open physes and the risk highest in the first two years	RTP is typically 10-12 months in children and adolescents. Return to pivoting and contact sport competitions should not be allowed until 14 months post-op especially in young children with open physes
Ithurburn et al., 2019 [13]	USA	Prospective cohort study	n=144, 9-25yo	Unspecified	ACL Reconstruction	Testing was done at RTP clearance and was the same for all groups, indicating all groups did rehabilitation for the same amount of time. Quad and hamstring muscle strength, limb symmetry index, self-reported knee function and knee functional performance, single hop, and crossover hop were all higher in the pediatrics group compared to the adolescent and young adult groups. 30% of pediatric population met all criteria cutoffs for RTS, higher than both adolescents and YA	Recommend development and use of more age specific rehab interventions depending on the needs of the patient populations and deficits they experience
Kay et al., 2018 [14]	USA	Systematic review and meta-analysis	n=20 studies, n=1156 ACL reconstructions, 6-19yo	Unspecified	ACL Reconstruction	92% of the pediatric population returned to sporting activities after ACLR and 81% returned to competitive levels of sport. However this was also associated with high graft rupture rate (13%) and injury to the contralateral ACL (14%). Less than half the studies evaluated reported specific criteria that patients were required to meet before returning to sports	Pediatric population had an increased risk of reinjury. Lack of specific criteria for patients to meet prior to RTP points to the need for universal guidelines for optimal rehabilitation and RTP protocols.
Lorange et al., 2024 [15]	Canada	Systematic review	n=24 studies, Number of patients unspecified, <18yo	Unspecified	ACL Reconstruction	Statistically significant risk factors for ACL re-injury included younger age and earlier RTS. Earlier RTS was a significant contributor to graft failure for combined timed and milestone-based RTS. Most common criteria in milestone groups were >90 limb symmetry using hamstring strength, quad strength and/or hop tests	Concluded that RTP should be delayed when possible in younger populations. Factors to assess when determining RTS ability and reinjury include skeletal maturity, surgical technique, biological factors in reinjury, and wound healing
Paterno et al., 2017 [16]	USA	Case-control and cohort	n=163, 10-27yo	Soccer, volleyball, basketball, football, and other	ACL Reconstruction	20% of subjects sustained a second ACL injury. Younger patients who present with moderate normalized triple hop for distance performance and greater limb asymmetry on the triple hop for distance test at the time of RTS, female patients, high self-reported confidence, normalized performance on the triple hop for distance >1.34 times body height, and greater limb symmetry are at greatest risk for a second ACL injury	Performance on the triple hop for distance test in both distance hopped and limb symmetry, sex, and self-reported knee confidence in young athletes can be used to determine who is at high risk for future ACL injuries after ACLR in the clinical setting
Pauw et al., 2023 [17]	Netherlands	Scoping review	n=63 studies, n=4456 patients, <18yo	Soccer, football, skiing, lacrosse, basketball, volleyball, gymnastics, rugby, ice hockey, and other	ACL Reconstruction	RTP after ACLR is not based on clear criteria. In 70%, even if RTS testing was performed, there were no consequences of the outcome of the test. Concluded that RTS testing should be part of a continuum	Testing to aid RTP decision-making should include the individual (biomechanics), task (sport type), and environment. Psychological monitoring is essential in this young population with knee injury.
Placella et al., 2015 [18]	Italy and the UK	Therapeutic study	n=24, 9-14yo	Soccer, volleyball, basketball, cycling, artistic dance, and fighting	ACL Reconstruction	With an all-inside ACL reconstruction, most players RTP on average at 6.43 months. Players in a professional club had a higher rate of return. There was no re-rupture in these patients but 10 of them had contralateral ACL rupture along with detailed differences in pre and post-operative measurements of the leg and strength in different ROMs	With an all-inside ACL reconstruction there is a good rate of return to sports at pre-injury levels or higher, high patient satisfaction, and a decent motor and proprioceptive function
Concussion							
Study	Country	Study Design	Patients	Sports Evaluated	Injuries Evaluated	Main Results	RTP Protocol Recommendations
Chrisman et al., 2019 [19]	USA	Prospective cohort study	n=863, 5-14yo	Football	Concussion	90% of subjects RTP by one month (mean 19.3). 90% had symptoms return to baseline by two months	RTP by one month in majority of patients
Haran et al., 2016 [20]	Australia	Prospective observational study	n=93, 5-18yo	Australian football, soccer, rugby, basketball and other	Concussion	42% not managed according to recommended guidelines for on-field management, 19% not immediately removed from play, 29% allowed to RTP the same day, 27% not assessed by qualified personnel, 7.9% allowed to RTP after <30 min. Overall, 72% were compliant with provided RTP guidelines	Many children with sports-related concussion are not formally assessed on-field and continue to play. RTP recommendations for children and adolescents will need to be modified from existing adult-focused guidelines to be more applicable
Johnson, 2012 [21]	Canada	Literature review	Unspecified	Football	Concussion	RTPs are important in the postconcussion period to avoid subsequent concussions or other damage, but they are less helpful in terms of prevention of first concussion	More effective concussion prevention is needed. Eliminating tackling from school football for youth under 16 is recommended to reduce rate of concussions
Lishchynsky et al., 2019 [22]	Canada	Prospective cohort study	n=30, 12-17yo	Ice hockey	Concussion	Median time in Moderate Vigorous Physical Activity (MVPA) as measured by accelerometer = 148.5 min. Groups were split into high and low activity (15 above 148.5 and 15 below). Group with high MVPA three days post-concussion took significantly more time to be medically cleared to RTP	Recommendations to limit the amount of time in MVPA initially for adolescent athletes may facilitate recovery following concussion
Marshall et al., 2018 [23]	Canada	Prospective cohort	n=759, 13-25yo	Ice hockey, football, soccer, rugby, basketball	Concussion	The Gapski-Goodman Test (GGT or modified GGT) was used as part of RTP decision-making to aid in final RTP clearance. Overall, female sex, younger age, and premorbid anxiety significantly increased the length of time between injury and RTP clearance. Although athletes were asymptomatic at the time of the test and had passed RTP protocols, 14.6% had an exacerbation of symptoms during the mGGT or GGT which could indicate they were not completely recovered. This test raises concerns about athletes underreporting symptoms to get RTP clearance and therefore putting them at higher risk for return of symptoms when they do return	Need to reexamine RTP protocols and use symptom provocation and dynamic physical exertion tests like the GGT to help inform RTP decisions. Also sport-specific symptom provocation tests are recommended as this test was designed for hockey and may be too cardiovascularly demanding for athletes whose sport does not require equal levels of fitness and therefore may have been at a disadvantage when taking the test
O'Brien et al., 2017 [24]	USA	Case control	n=217, <18yo	Ice hockey, football, soccer, basketball, and other	Concussion	12% of patients who were symptom-free at rest had recurrence of symptoms upon resuming exercise. Higher risk of recurrence was in patients who did not lose consciousness during injury, had history of undiagnosed concussion, and who had longer symptom duration	The identified risk factors should be considered when the treating clinician is determining the pace at which an athlete should progress through stepwise activity-resumption protocols
Purcell, 2009 [25]	UK	Literature review	n > 60 studies, <18yo	Unspecified	Concussion	Younger children take longer to recover and there is less information about the topic in younger children. Most guidelines are adapted from adult guidelines	Patients should not return to sports until asymptomatic and anticipate younger children taking longer to recover
Seehusen et al., 2021 [26]	USA	Observational prospective cohort study	n=32, 12-18yo	Unspecified	Concussion	Activity levels were measured and split into two groups: those who took greater than and less than 28 days to RTP. Patients with >28 days to RTP took fewer steps per day, exercised fewer days per week, and fewer total minutes per week. However, those who returned to play <28 days had a lower (but not significant) mean Post Concussion Symptom Inventory (PCSI) rating at the RTP visit and had a lower symptom burden initially post injury	Suggests that exercise and activity may help symptoms resolve sooner and decreased time to RTP
Jildeh et al., 2020 [27]	USA	Literature review	n= 357, 14-18yo	Football, hockey, soccer	Concussion	Female players with a history of concussion and those diagnosed in the clinic rather than in the game had significantly increased RTS. Memory imPACT scores increased with recurrent concussions and visual and motor speed reaction time scores decreased with recurrent concussions	ImPACT score used to quantify athlete readiness to return
Upper Extremity Protocols							
Study	Country	Study Design	Patients	Sports Evaluated	Injuries Evaluated	Main Results	RTP Protocol Recommendations
Cain et al., 2021 [28]	USA	Case series	n=29, <17yo, male	Overhead sports athletes: Baseball (23), football quarterbacks (3), tennis (1)	ORIF for displaced medial epicondyle fracture	97% of overhead athletes returned to their previous level of sports. Mean self-reported fastball velocity increased significantly for pitchers from 76 mph pre-injury to 83 mph post injury. Mean satisfaction score of 9.8. Fracture union rate was 96%.	ORIF of medial epicondyle fractures can result in reliable, pain-free return to competitive sports: on average, seven month recovery for RTP
Manoukov et al., 2024 [29]	France	Retrospective review	n=17, 12-17yo	Soccer, judo, horseback riding, motocross, rugby, handball, rollerblading	Traumatic complete rotator cuff tear	Complete RTC tears are uncommon in children but should be considered as a differential diagnosis in cases of combat or contact sports with residual post-traumatic pain. 82.4% (14/17) returned to sport at their pre-injury level, suggesting surgery is the appropriate treatment option for full-thickness tears	In more than 80% of cases, the surgical repair enabled the patient to return to sport. However, the mobility assessment of the operated shoulder performed several months after repair was incomplete and/or imprecise in some cases
Patel et al., 2024 [30]	USA	Literature review	Unspecified	Baseball, gymnastics and other	Upper extremity musculoskeletal injuries	Sport specialization before puberty may be an independent risk factor for overuse injuries and therefore a need to establish guidelines for overuse injuries and return to sport. Overuse injuries recur more frequently than acute injuries and more research on physeal injuries is required	Until more specific guidelines are solidified for RTP for physeal injuries, joint decision-making among the provider, caregiver, and patient is paramount
Sakata et al., 2020 [31]	Japan	Case control	n=81, < or = 12yo	Baseball	Capitellar osteochondritis dissecans (capitellar OCD)	All patients in the study received rehab treatment initially and the overall RTP rate of non-operative treatment was 70.4%. Rehab program consisted of: (1) full ROM exercise permitted with non-painful ROM, including thoracic and pericapsular programs. Goal was to obtain full extension of the elbow with no pain in four weeks before starting, (2) flexor- pronator muscle strength training. Then progressed to (3) closed kinetic chain exercises, then (4) interval throwing program. Successful RTP was defined as RTP at the same position as before injury with no pain for one season	For early-stage capitellar OCD there was a four times increased success rate with OCD rehabilitation and non-operative treatment. Patients were allowed to RTP after asymptomatic completion of interval throwing program
Zaremski et al., 2017 [32]	UK	Systematic review and meta-analysis	n=17 studies, n=654 shoulder instability events, <18yo	Unspecified contact and non-contact sports	Shoulder subluxation or dislocation	RTP rates were higher for primary operative patients versus primary non-operative and secondary operative	Primary operative treatment had the highest rate of full RTP with the least rate of recurrence
Lower Extremity Protocols							
Study	Country	Study Design	Patients	Sports Evaluated	Injuries Evaluated	Main Results	RTP Protocol Recommendations
Eberbach et al., 2021 [33]	Germany	Prospective cohort study	n=14, 11-22yo	Soccer	>6 weeks pubic related groin pain	Pubic related groin pain is an overuse injury with an average RTP period of 135.3 +/- 83.9 days. All 14 players returned to sport with negative pubic related groin pain, pain of the RA and adductor insertions at pubic bone, pain during active adduction in adductor squeeze tests, groin pain during or after playing soccer and reduction in Tegner activity level due to groin problems	A six level stepwise rehab program is recommended when treating pubic related groin pain. Expect longer RTP with younger patients and those with more severe bone marrow edema
Martinez-Silvan et al., 2023 [34]	Qatar and South Africa	Prospective collection of injury data	n=85, 11-19yo	Soccer and track and field	Lower extremity muscle injuries	Three muscle groups had the largest injury counts: hamstring, adductor, and quads. For hamstrings, injuries involving the tendon had significantly greater time to RTS than compared with myotendinous junction and muscle. MTJ had a significantly greater time to RTP than muscle. Proximal hamstring injuries had greater time to RTS than mid portion and distal	Different times to RTS are expected depending on the sport, affected muscle, and specific injury characteristics, such as location, involved structure, and grade
Migliorini et al., 2022 [35]	Germany, Italy, UK	Systematic review	n=15 studies, n=477 patients,<18 yo	Unspecified	MPFL reconstruction	Measured clinical outcomes, return to sport and complications. All patient-reported outcomes measures (PROMs) were significantly improved at the last follow-up. Complications: 5% experienced further dislocations, and 2% experienced subluxations during the follow-up period	Autograft MPFL reconstruction in skeletally immature patients with recurrent patellar instability is a treatment option with significant clinical improvements. The mean time to return to sport was 6.1 ± 1.1 months. Of the included patients, 27% had reduced their level of activity, while 87% returned to their previous level of sport
Rueth et al., 2023 [36]	Germany	Retrospective study	n=101, <18yo	Unspecified	Acute patellofemoral dislocation	Anatomic MPFL reconstruction using the gracilis tendon allograft is safe in children and adolescents with low risk of recurrent instability. 84% of patients were satisfied with their surgical result, 86.6% returned to sports. Versus conservative management which has a recurrent instability of 38-71%. Kujala score improved significantly from 47.1 pre-op to 85.2 post op (p<0.01)	MPFL reconstruction with gracilis is associated with increased return to sports compared to conservative treatment which has increased likelihood of recurrent instability
Saper et al., 2019 [37]	USA	Case series	n=28, 12-16yo	Unspecified	Patellofemoral instability injuries	Surgeons should not solely rely on timing to allow RTP but use strength and function testing as well. Deficits were especially seen in isometric quadriceps strength in the affected limb	Patients were not ready to RTP by seven months and may require rehab programs longer than eight months to achieve adequate strength and functional stability
Stallone et al., 2020 [38]	Italy	Retrospective study	n=36 completed questionnaires and n=23 tested with arthrometers, <18yo	Skiing/snowboarding, motocross, soccer, basketball, biking, volleyball	Avulsion fracture of the tibial spine	78% of patients returned to sport at their previous activity level (eight amateur, 13 competitive, seven elite). Objective knee stability was restored in 90% of cases, however 22% of children did not return to sports due to referred pain or fear of reinjury. It couldn't be determined whether this was due to mechanical problems or psychological issues as there was no difference in knee laxity or knee motion between children who RTP and those that chose not to	If properly treated, pediatric avulsion fractures of the tibial eminence achieve a high rate of successful healing, with complete restoration of knee stability and in most cases of function, and early return to sport activities at a pre-injury level. Despite this, many children stopped playing sports, mainly because fear of reinjury
Vadhera et al., 2022 [39]	USA	Systematic review	n=90, <18yo	Soccer, sprinting, gymnastics, football	Apophyseal avulsion fractures of the ischial tuberosity (AFIT)	82% reported successful RTS at avg 7.0 +/- 5 months. Soccer players had the highest RTS rate of 88%. Surgical treatment also had lower complications (16%) than those treated non-operative (33%)	Athletes who underwent surgery had a significantly higher RTS rate of 94.7% compared with non-operative. Athletes with initial misdiagnosis had an RTS of 8.2 +5.7 months whereas those diagnosed accurately on initial presentation returned at a mean of 6.8 +5.0 months
Weber et al., 2020 [40]	USA	Case series	n=49, collegiate-level athletes, average age 19yo	Cutting sports (Soccer, basketball, lacrosse, and field hockey), flexibility sports (Dance, gymnastics, figure skating, yoga, and martial arts), impingement sports (Hockey, crew, water polo, baseball catcher, and breaststroke swimmer), asymmetric/overhead sports (Baseball, softball, tennis, golf, and volleyball), and endurance sports (Track, cross-country, other running, cycling, and swimming [other than breaststroke])	Femoroacetabular impingement syndrome (FAI)	Collegiate-level athletes RTP at a higher and more predictable level. Return to sport time average of 1.98 +/- 0.94 years. Overall return to sport rate was 89.7%. No difference in RTS rates except in endurance athletes with a lower rate at 66.6%	All athletes followed the standard rehab protocol of a 24-week program with five phases. Specific adjustments for each phase were made for cardiovascular activity, hip ROM and WB status of lower extremity
Spine Protocols							
Study	Country	Study Design	Patients	Sports Evaluated	Injuries Evaluated	Main Results	RTP Protocol Recommendations
Kasamasu et al., 2022 [41]	Japan	Retrospective study	n=180, 6-18yo	Baseball, soccer, track and field, basketball, tennis and other	Lumbar spondylolysis	Patients treated with either trunk brace and cessation of sports activity or treated for pain only. RTS was 98.9% at 4.7 months in the bracing group and 97.6% at 1.8 months in the pain management group. Mean RTS was longer in bracing group due to brace therapy and confirmation of bone healing. Rehabilitation program was recommended and 88.7% of patients completed the program. Importance of PT in return to sport after spondylolysis has shown to be important but details are not clear	Main rehabilitation strategies recommended included mobilization of thoracic spine, stretching of hamstring and quads and core muscle training. Authors attributed low recurrence rates to the rehabilitation program

*Risk of Bias Assessment*

Given the heterogeneity of included study designs, a formal, validated risk-of-bias tool was not applied. Study quality and methodological limitations were assessed descriptively, including evaluation of study design, sample size, follow-up duration, and clarity of RTP criteria.

Protocol Registration

This systematic review was not prospectively registered in PROSPERO or any other publicly accessible registry.

Results

**Figure 2:**
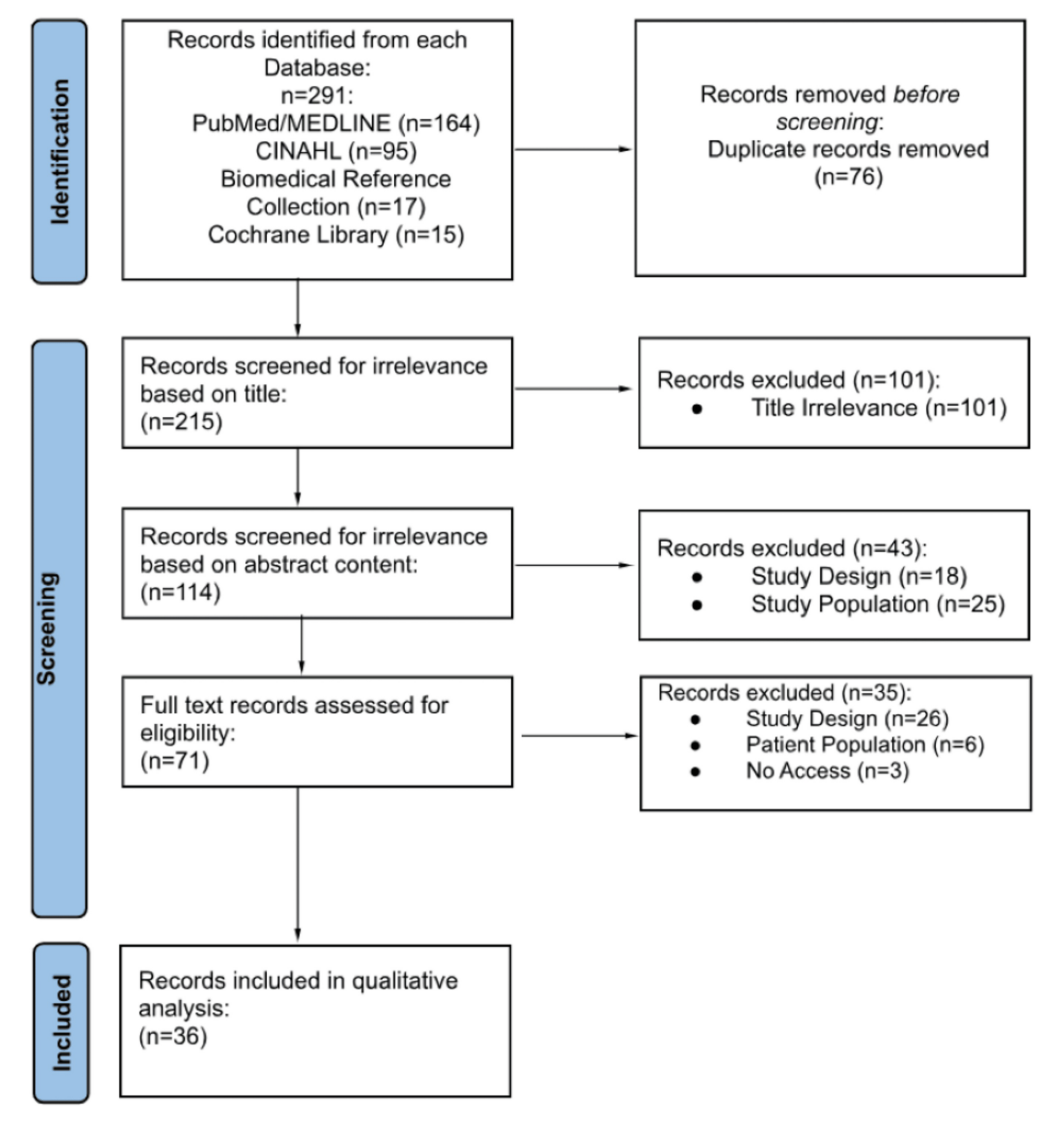
PRISMA flow diagram for article inclusion and exclusion PRISMA: Preferred Reporting Items for Systematic Reviews and Meta-Analyses

This study followed the PRISMA process of article inclusion and exclusion as seen in Figure 2. In total, 291 potential articles were identified from the primary literature search performed. Following screening and selection, a total of 36 articles were included for this review. Outcome measures extracted from these full-text records include publication date, country of origin, study design, patient demographics, sports evaluated, injuries evaluated, main results, and RTP protocol recommendation.

Study Characteristics

The studies included in this analysis originated from a total of 11 nations, with the top two countries being the United States (36%, n=13) and Canada (11%, n=4), followed by the Netherlands, Germany, the United Kingdom, France, Italy, Australia, Japan, Qatar, and South Africa. Study sample sizes ranged from n=14 to n=4456, with an average study size of 321.8 patients. Studies were published between 2009 and 2024, with half of the studies being published within the last five years.

Study designs of the final articles included five case series, five prospective cohort studies, four retrospective studies, four literature reviews, three systematic reviews, two case control studies, two retrospective reviews, two scoping reviews, two prospective observational studies, two systematic reviews and meta analysis, one retrospective cross sectional, one multicenter retrospective cohort, one prospective collection of injury data, one case control and cohort, and one therapeutic study.

Of the included studies, there were 13 ACL reconstruction protocols (36%), nine concussion protocols (25%), and one protocol for each of the following: open reduction and internal fixation (ORIF) for displaced medial epicondyle fractures (2.7%), greater than six weeks pubic-related groin pain (2.7%), lumbar spondylolysis (2.7%), traumatic complete rotator cuff tear (2.7%), medial patellofemoral ligament (MPFL) reconstruction (2.7%), muscle injuries (2.7%), upper extremity musculoskeletal injuries (2.7%), acute patellofemoral dislocation (2.7%), capitellar osteochondritis dissecans (OCD) (2.7%), patellofemoral instability injuries (2.7%), avulsion fracture of the tibial spine (2.7%), apophyseal avulsion fractures of the ischial tuberosity (AFIT) (2.7%), femoroacetabular impingement syndrome (FAIS) (2.7%), and shoulder subluxation and dislocation (2.7%).

The sports that were analyzed in this review included soccer, football, basketball, volleyball, baseball/softball, tennis, lacrosse, skiing, rugby, ice hockey, track and field, judo, horseback riding, motocross, rollerblading, gymnastics, dance, cycling, fighting, handball, and unspecified. Soccer made up 47.2% (n=17) of the articles analyzed. Followed by football and basketball (33.3%, n=12), baseball/softball and volleyball (19.4%, n=7), ice hockey (16.6%, n=6), rugby (13.8%, n=5), lacrosse and track and field (11.1%, n=4), tennis, skiing, cycling, gymnastics, and martial arts (8.3%, n=3), dance, and motocross (5.5%, n=2), horseback riding, rollerblading, handball (2.7%, n=1). A total of 36.1% (n=13) of the articles did not specify which sports were analyzed.

Discussion

RTP protocols are necessary to ensure a safe and efficient return to activities, especially in the pediatric population. Protocols vary based on sport, age, type of injury, and the invasiveness of the treatment option chosen. Further standardization in this field will increase applicability and understanding among the patient, parent, physician, and coach relationship.

The most common sport researched based on our inclusion criteria was soccer, followed by football and basketball. Many studies also evaluated athletes near the time of physeal closure. During puberty and the associated growth spurts, bone ossification lags behind linear growth and may increase the injury risk for a pediatric athlete [28]. Many of the protocols reviewed recommended increased caution and prolonged return to full activity in the pediatric population, given their increased risk of reinjury [9,14,15,25].

ACL Reconstruction Protocols

A majority of the pediatric sport injury research included in this review focused on the treatment of ACL tears (Table 1). Four out of 13 articles discussing RTP after ACL reconstruction recommended full RTP between nine and 12 months, with one strongly cautioning RTP before six months post-operatively [7,8,10,12]. In younger patient populations, time to RTP should be delayed longer due to open physes and increased risk of reinjury. Geffroy et al. recommended that children with open physes not return to sport until 14 months after the injury [12]. It was also noted that in the pediatric population, age and sex may have no clinically important effect on physical therapy (PT) visit utilization, time between surgery and RTP test completion, or hop test performance [7].

One protocol recommended assessing readiness to return at three and six months post-operatively. They found that increased quadriceps strength at both assessments was associated with increased self-readiness and earlier functional RTP [6]. Many articles recommended sports-specific exercise and rehabilitation programs tailored to the pediatric athlete [8,11,13]. There has also been a trend in increased use of activity-based and biomechanical protocols as opposed to the traditional time-based guidelines. Functional and strength evaluations, as well as biomechanical metrics, are used in many protocols to determine an athlete’s readiness to return to sport. Dietvorst et al. recommended that RTP guidelines be created around these metrics using age-specific normal values due to the variability in muscle strength and proprioception in the pediatric population. They recommended RTP testing for patients aged 16-18, including quadriceps and hamstring strength testing, hop tests, movement quality assessment during sport-specific tasks, limb symmetry measurements, and patient-reported outcome measures (PROMs) [11]. RTP testing for patients aged 12-16 should include hop tests, movement quality, and pediatric PROMs. In patients <12 years old, less emphasis should be placed on muscle strength and hypertrophy, and only pediatric PROMs should be used [11].

Assessing the risk of reinjury should also be included in RTP guidelines, with one study aiming to use data from evaluations, such as the triple hop test, and other risk factors, like age, to predict the risk of ACL reinjury after surgical reconstruction [16].

Concussion Protocols

Studies on concussions in pediatric athletes demonstrated the need for protocols tailored specifically to the pediatric population, rather than modifications of adult guidelines [20,25]. They also recommended increased use of quantitative and sport-specific testing in addition to risk factor stratification when planning for return to sports [23,24,27]. These include the Gapski-Goodman Test (GGT or modified GGT), sport-specific symptom provocation tests, and ImPACT scores. Haran et al. advocated for increased formal on-field assessment of pediatric athletes to avoid continuing play with a concussion, which can lead to prolonged return after diagnosis [20]. Purcell found that younger children took longer to recover from concussions than adults, highlighting the importance of incorporating this information into age-specific guidelines provided to clinicians who are evaluating and treating pediatric patients [25]. Johnson advocated for primary prevention of concussions in the pediatric population by limiting the age of contact in sports, such as no tackling in football before the age of 16 [21].

Concussion evaluation has the added complication of patients underreporting their symptoms to obtain earlier RTP clearance. Including physical exertion tests that are modified for the patient’s specific sport, such as the GGT or modified GGT, may help evaluate patients more objectively when determining their ability to RTP after a concussion [23].

Upper Extremity Protocols: ORIF for Medial Epicondyle Fracture, Traumatic Complete Rotator Cuff Tear, Upper Extremity Musculoskeletal Injuries, Capitellar OCD, Shoulder Subluxation and Dislocation

While fewer articles discussed upper extremity injuries, their findings are extremely relevant to many pediatric sports. Many authors recommended interval throwing programs as part of rehabilitation for upper extremity injuries in overhead youth throwing athletes [28,30,31]. Following medial epicondyle ORIF, Cain et al. recommended a PT protocol that starts with placing the upper extremity in a posterior splint for the first five days after surgery for a medial epicondyle ORIF. At one week, the patient begins with passive and active range of motion as well as light strengthening for the shoulder and scapula. Light strengthening exercises are initiated at week three, and a full passive range of motion is encouraged at eight weeks. If the fracture is healed, the patient begins more aggressive strengthening at eight weeks and light throwing at 16 weeks. Patients in this study were allowed to return to sports six months after surgery. This post-operative rehabilitation program was also documented to not only help athletes return to sport, but also increase pitching velocity above their pre-injury level per patient reports [28].

After surgery for medial epicondyle fractures and traumatic rotator cuff tears, studies found that 97% and 82.4% of athletes returned to their pre-injury level of sports, respectively [28,29]. In addition, Zaremski et al. found the highest RTP rates were in patients who pursued primary operative treatment for shoulder subluxations and dislocations compared to those who initially pursued nonoperative treatment followed by operative management [32]. This suggests that early surgical stabilization can be an appropriate first-line treatment for pediatric populations due to their increased risk of recurrence. An additional area of concern in overhead or throwing athletes is overuse injuries at a younger age. Patel et al. reported that sport specialization before puberty may be an independent risk factor for these injuries and advocated for the development of standardized protocols to avoid repetitive strain and overload [30].

Lower Extremity Protocols (Excluding ACL Reconstruction): Pubic-Related Groin Pain, Lower Extremity Muscle Injuries, MPFL Reconstruction, Patellofemoral Dislocation, Patellofemoral Instability, Avulsion of the Tibial Spine, Apophyseal Avulsion Fracture of the Ischial Tuberosity, FAIS

While many of the lower extremity injury articles analyzed were about ACL reconstruction, there was also a significant number that addressed other lower extremity pathologies in the pediatric population. Pubic-related groin injuries are a common overuse condition that occurs in the lower extremity. A stepwise rehab program consisting of six levels is recommended. The program begins with stopping all sports activities and emphasizes lymphatic drainage and vitamin D supplementation. Next, it recommends progression through adductor strengthening, core stability, and aerobic exercise, ending with preventative strengthening twice a week. Each level has an assessment that confirms safe progression to the next. This study also places emphasis on the expectation of a longer RTP with younger patients [33]. One study focused on college-aged athletes with femoroacetabular impingement (FAI) and discussed that RTP was higher and more predictable in older athletes compared to their younger counterparts [40].

Approximately 87% of patients returned to their previous level of sport after MPFL reconstruction surgery. Migliorini et al. reported the mean time to return was 6.1 ± 1.1 months, while Rueth et al. found patients returned to sport within two years [35,36]. A retrospective review of adolescent patients who underwent MPFL reconstruction by Saper et al. used strength and functional return to sport testing and found these athletes may need prolonged rehab beyond eight months for proper recovery [37]. This emphasizes the transition away from time-based guidelines and toward functional and sport-specific protocols to ensure optimal rehabilitation.

In a systematic review, Vadhera et al. reported that athletes under 18 years who underwent surgical repair for apophyseal AFIT had a significantly higher RTP rate (94.7%) compared to those who were treated nonoperatively (73.1%). They also found the incidence of treatment-related complications was lower in athletes who underwent surgical management (16%) compared to those treated nonoperatively (33%) [39]. Their review highlights that early indicated surgery is a reasonable treatment option for patients who desire the highest chance of returning to their athletic activities.

Psychological Impact and Considerations

The role of psychological interventions in postoperative rehabilitation has been largely unexplored, but their potential benefits have recently gained recognition. Many studies found that for athletes who chose not to return to sport after injury, their decision was largely due to fear or the psychological stress of reinjury [8,38,42]. This has led to advocacy for the addition of early psychologist involvement to set expectations throughout the duration of care. Paterno et al. and Pauw et al. recommended assessing psychological readiness and athlete confidence prior to RTP in pediatric patients [16,17]. In adults, studies have found that anxiety about reinjury and lack of confidence in the injured knee have affected rehabilitation outcomes after ACL reconstruction [42]. A large systematic review and meta-analysis evaluating 48 studies and 5770 participants with an average age of 25.1 years found that despite successful outcomes on knee-impairment function tests, fear of reinjury was the most common reason to not return to the previous level of sport or resume sports following ACL surgery [43]. Due to this discrepancy between the objective function of the knee and the decision not to return to sports, it has been suggested that psychological factors may also contribute to decreased RTS for pediatric athletes. Studies have shown a relationship between pediatric patients who perceived having a knee disability and decreased self-esteem, mental health concerns, and impaired emotional functioning [17,42]. Athletes with higher levels of psychological readiness to RTP, greater motivation to RTP, and higher self-efficacy of knee function were shown to successfully return to their previous level of sport, but most RTP protocols do not include evaluations of these metrics [44].

Experiencing a significant injury and subsequently undergoing surgery and rehabilitation can be an arduous process for anyone, but for the pediatric population, this experience may be more heavily influenced by psychological factors. Many studies are encouraging sports psychologists and mental health professionals to intervene early in the rehabilitation process and continue evaluations throughout recovery to help improve psychological readiness to play [16,17,42]. A multidisciplinary approach that combines physical and non-PTs may be an effective strategy for enhancing surgical outcomes and athletes’ ability to return to sport.

Limitations

This review has several limitations. The included studies demonstrated heterogeneity in injury type, study design, RTP definitions, and outcome reporting, which limited direct comparison and precluded quantitative meta-analysis. RTP criteria were inconsistently defined across studies, reflecting the absence of standardized pediatric frameworks. Additionally, the inclusion of athletes under 25 years of age may extend beyond strict skeletal immaturity; however, this approach was selected to reflect the structure of the available literature, which frequently groups adolescent and young adult athletes together. A formal, validated risk-of-bias instrument was not applied, which may limit structured assessment of study quality across included studies. Finally, many included studies were observational in design, limiting the overall strength of evidence.

## Conclusions

While our review evaluates a variety of studies and methods of determining RTP, a common theme among them is the necessity of standardized, yet patient-oriented guidelines for the pediatric population’s most common injuries. RTP protocols for each sport can prevent confusion and tension in the decision-making process. Additionally, a majority of the studies discussed the need for functional exercises and sport-specific testing as the athlete advances through the phases of recovery. Emphasis on reinjury risk and the psychological impact of an injury on return to preinjury sports participation are also vital to the mental and physical health of the young athlete, and should be further explored when developing standardized frameworks. More studies must be conducted in pediatric populations as opposed to adapting adult protocols, given their differences in anatomy, skeletal development, rigor of play, skills, and social implications. We hope that this review sheds light on the variety of research being done on pediatric RTP protocols and contributes to the development of safe best practices for the adolescent athletic population.
